# Religion and health: not always good

**DOI:** 10.31744/einstein_journal/2020CE6133

**Published:** 2020-11-25

**Authors:** Jacyr Pasternak

**Affiliations:** 1 Hospital Israelita Albert Einstein São PauloSP Brazil Hospital Israelita Albert Einstein, São Paulo, SP, Brazil.

Dear Editor,

Akerman et al.,^(^^1^^)^ allude to possible usefulness of religious beliefs toward health. There were 69,333 publications on this subject indexed in PubMed® as by Aug 26, 2020, and the hard evidence for health gains to persons with religious convictions is not overwhelming, to say the least. According to Levin there are hints of association, but none that shows that the possible health gains are casual.^(^^2^^)^ Papers exist suggesting that religion is a protective factor for health. Some are funded by Templeton, with a well-known bias for religion, so there are conflicts of interest on board.^(^^3^^)^ On the other hand, there are studies on the risks of infections associated with religious rituals, and they are real and documented.^(^^4^^)^ There are other instances of unhealthy facts associated with religion:

– The refuse of Jehovah's witness to blood transfusion.
– The refuse of medical care by Christian Science, that considers all diseases linked to lack of faith.
– The propaganda of Islamic Imams against polio vaccination, considered to be a diabolical plan of Western governments to decrease Muslin women fertility.
– Faith healing by evangelical ministers in religious ceremonies, and later the perception by those cured that they are not cured at all.
– Pseudoscientific health practices, such as Homeopathy, based on one (just one) non-randomized and non-blind study by Hanneman centuries ago, so faith-based.

There are religious like attitudes, as the existence of groups like the anti-vaxxers that are based on a known and fraudulent study published by Wakefield in The Lancet.^(^^5^^)^ Wakefield et al., did not use religion as a part of his fraud, but people who really believe in the association between autism and measles vaccine use this known fraudulent study as some type of divine revelation, and they defend it against all evidences and facts available.

The point is: religion and religious like attitudes can be bad for your health.
