# Supplementary material for: Religion and health: not always good
Source: Einstein (Sao Paulo). 2020 Nov 25;18:eCE6133. doi: 10.31744/einstein_journal/2020CE6133 (PMC7690927; doi:10.31744/einstein_journal/2020CE6133)
Supplement: Translation

# Translation: Religião e saúde: nem sempre é bom

**Authors:** Jacyr Pasternak

**Affiliations:** 1 São PauloSP Brasil Hospital Israelita Albert Einstein, São Paulo, SP, Brasil.

Caro Editor,

Akerman et al.,^(^^1^^)^apontam para a possível utilidade das crenças religiosas para a saúde. Até 26 de agosto de 2020, cerca de 69.333 publicações sobre o assunto estavam indexadas no PubMed®. A evidência dos ganhos para saúde dos indivíduos com convicções religiosas não é tão extensa. De acordo com Levin, há sinais de associação, mas nenhum deles mostra que os possíveis ganhos na saúde são casuais.^(^^2^^)^Há artigos sugerindo que a religião é um fator protetivo para existência da saúde. Alguns estudos são financiados por Templeton, com conhecido viés religioso, havendo, portanto, conflitos de interesse na questão.^(^^3^^)^Por outro lado, há estudos sobre riscos de infecções associadas aos rituais religiosos – esses reais e documentados.^(^^4^^)^Há outros exemplos de fatores prejudiciais que estão associados à religião:

– A recusa das testemunhas de Jeová à transfusão de sangue.
– A recusa do cuidado médico pela Ciência Cristã, que considera que toda doença está associada à falta de fé.
– A propaganda do imame desfavorável à vacinação contra a pólio, considerada um plano diabólico de governantes ocidentais para reduzir a fertilidade das mulheres islâmicas.
– A cura pela fé propagada por ministros evangélicos em cerimônias públicas e a percepção tardia daquele que foi curado de que não está curado.
– As práticas pseudocientíficas de saúde, como a homeopatia, baseada em um (apenas um) estudo não randomizado e não cego conduzido por Hanneman há séculos atrás e, portanto, baseado na fé.

Há religiões como atitudes, por exemplo, a existência de grupos como o movimento antivacina, inspirado em estudo conhecido e fraudulento publicado por Wakefield no The Lancet.^(^^5^^)^Wakefield et al., não utilizaram religiosidade como parte da fraude, porém as pessoas que realmente acreditam na associação entre autismo e vacina do sarampo consideram esse estudo um tipo de revelação religiosa e o defendem como incontestável, apesar de toda evidência e dos fatos que existem.

O ponto é: religião e religiosidade, como uma atitude, podem ser prejudiciais à saúde.
